# Intrafamilial Phenotypical Variability Linked to PRKAG2 Mutation—Family Case Report and Review of the Literature

**DOI:** 10.3390/life12122136

**Published:** 2022-12-18

**Authors:** Andreea Sorina Marcu, Radu Vătăşescu, Sebastian Onciul, Viorica Rădoi, Ruxandra Jurcuţ

**Affiliations:** 1 Expert Center for Genetic Cardiovascular Diseases, Emergency Institute for Cardiovascular Diseases, 258 Fundeni Street, 022328 Bucharest, Romania; 2 Cardiology Department, Emergency Clinical County Hospital Craiova, 1 Tabaci Street, 200642 Craiova, Romania; 3 Department of Cardiology, University of Medicine and Pharmacy Craiova, 2 Petru Rares Street, 200349 Craiova, Romania; 4 Cardiology Department, Emergency Clinical Hospital Floreasca, 8 Calea Floreasca, 014461 Bucharest, Romania; 5 Department of Cardiothoracic Pathology, University of Medicine and Pharmacy Carol Davila, 8 Eroii Sanitari Blvd., 050474 Bucharest, Romania; 6 Emerald Medical Center, 75 Nicolae G. Caramfil Street, 077190 Bucharest, Romania; 7 Department of Medical Genetics, University of Medicine and Pharmacy Carol Davila, 8 Eroii Sanitari Blvd., 050474 Bucharest, Romania

**Keywords:** hypertrophic cardiomyopathy, genocopy, PRKAG2, Wolf–Parkinson–White syndrome

## Abstract

PRKAG2 syndrome (PS) is a rare, early-onset autosomal dominant phenocopy of sarcomeric hypertrophic cardiomyopathy (HCM), that mainly presents with ventricular pre-excitation, cardiac hypertrophy and progressive conduction system degeneration. Its natural course, treatment and prognosis are significantly different from sarcomeric HCM. The clinical phenotypes of PRKAG2 syndrome often overlap with HCM due to sarcomere protein mutations, causing this condition to be frequently misdiagnosed. The syndrome is caused by mutations in the gene encoding for the γ2 regulatory subunit (*PRKAG2*) of 5′ Adenosine Monophosphate-Activated Protein Kinase (AMPK), an enzyme that modulates glucose uptake and glycolysis. *PRKAG2* mutations (OMIM#602743) are responsible for structural changes of AMPK, leading to an impaired myocyte glucidic uptake, and finally causing storage cardiomyopathy. We describe the clinical and investigative findings in a family with several affected members (NM_016203.4:c.905G>A or p.(Arg302Gln), heterozygous), highlighting the various phenotypes even in the same family, and the utility of genetic testing in diagnosing PS. The particularity of this family case is represented by the fact that the index patient was diagnosed at age 16 with cardiac hypertrophy and ventricular pre-excitation while his mother, by age 42, only had Wolff–Parkinson–White syndrome, without left ventricle hypertrophy. Both the grandmother and the great-grandmother underwent pacemaker implantation at a young age because of conduction abnormalities. Making the distinction between PS and sarcomeric HCM is actionable, given the early-onset of the disease, the numerous life-threatening consequences and the high rate of conduction disorders. In patients who exhibit cardiac hypertrophy coexisting with ventricular pre-excitation, genetic screening for *PRKAG2* mutations should be considered.

## 1. Background

PRKAG2 syndrome (PS) is a rare, early-onset autosomal dominant, inherited disease, clinically characterized by ventricular pre-excitation, cardiac hypertrophy and progressive conduction system degeneration [1]. The syndrome is caused by mutations in the gene encoding for the γ2 regulatory subunit (*PRKAG2*) of 5′ Adenosine Monophosphate-Activated Protein Kinase (AMPK) [2], an enzyme that modulates glucose uptake and glycolysis [3]. Affected individuals develop ventricular wall thickening (6) which is not due to myocyte hypertrophy, disarray and fibrosis, as defines sarcomeric hypertrophic cardiomyopathy (HCM), but instead is characterized by an increased intracellular glycogen deposition in myocytes [4].

The prevalence of PRKAG2 syndrome is 0.23–1% in patients with suspected HCM [5], but it may prove higher because of the larger availability of genetic testing for HCM worldwide. PS onset of symptoms frequently occurs within the first three decades of age [6] and involves numerous life-threatening events; therefore, early identification, adequate risk stratification and prolonged monitoring is required.

## 2. Case Presentation

**Figure 1:**
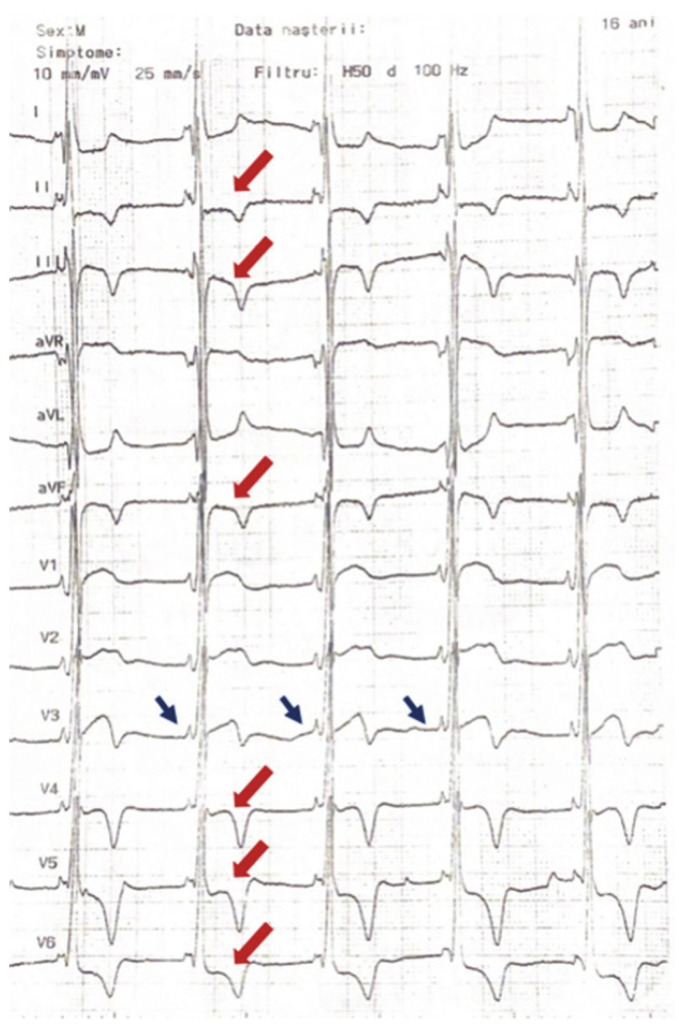
Resting electrocardiogram of the young patient shows a short PR interval 90 ms (blue arrow), bi-atrial enlargement markedly increased left ventricle voltages (Sokolow–Lyon index of 38 mm) with inverted T waves seen in the inferior and lateral leads (red arrow).

We describe the case of an asymptomatic adolescent male 16 years old referred for further evaluation after incidental electrocardiographic (ECG) findings consisting of a short PR interval, bi-atrial enlargement and criteria for left ventricle hypertrophy (LVH) (Figure 1). He was also recently diagnosed with severe hypertension. There was no other known past medical history.

**Figure 2:**
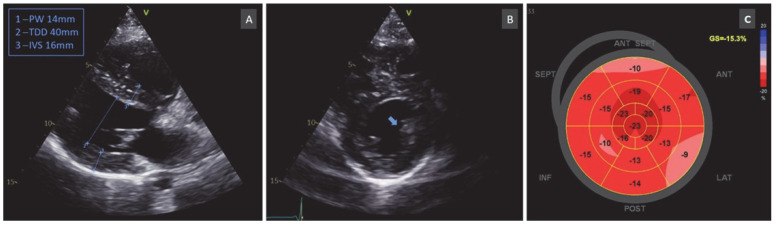
Transthoracic 2D echocardiography: (**A**) Parasternal long-axis section showing concentric hypertrophy involving mainly the basal septum (diastolic interventricular septum thickness of 16 mm). (**B**) Parasternal midventricular short axis revealing a prominent anterolateral papillary muscle (blue arrow). (**C**) Bull’s eye of LV myocardial deformation showing abnormal values of a global longitudinal strain of −5.3%.

Two-dimensional echocardiography of the proband confirmed concentric biventricular hypertrophy (with a relative wall thickness of 0.7, interventricular septum of 16 mm, posterior wall of 14 mm, and right ventricle free wall thickness of 7 mm) with a hypertrophied anterolateral papillary muscle and thickening of the ventricular apex with obliteration at end-systole (with a maximum wall thickness of 14 mm circumferentially at the apical level) (Figure 2), a good systolic function (normal left ventricle ejection fraction), but an altered longitudinal dysfunction (borderline low systolic myocardial velocities and low global longitudinal strain). The patient did not present dynamic obstruction of the left ventricle (LV) outflow tract.

A 24 h ambulatory ECG monitor showed a normal sinus rhythm with a short period of idioventricular rhythm (four QRS complexes). Additionally, ambulatory blood pressure (BP) recording was performed for 24 h showing arterial hypertension: a diurnal average BP 153/80 mmHg, a nocturnal average BP 146/77 mmHg (a reduced dipping BP pattern), and a maximum diurnal BP 184/99 mmHg. The patient was maintained on daily 5 mg of amlodipine, achieving good blood pressure control.

**Figure 3:**
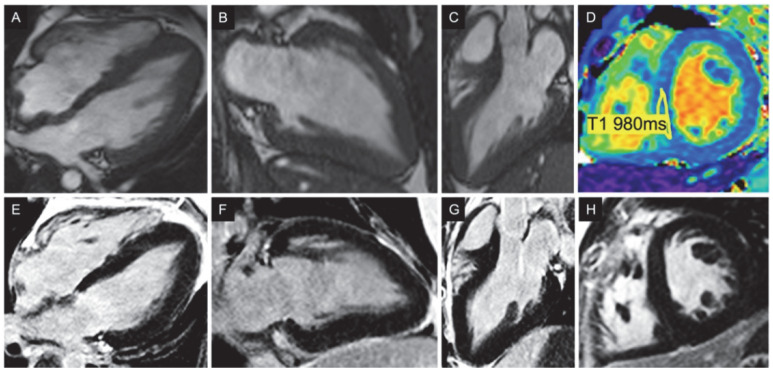
Contrast enhanced cardiovascular magnetic resonance. (**A**–**C**) Diastolic frames of cine images in 4, 2 and 3 chambers, respectively, showing a relative increase of left ventricular (LV) wall thickness at the apex compared with the base, with a typical ace-of-spades LV configuration. Although the maximum thickness of the apical walls is only 13 mm, there is a striking loss of normal thinning of the LV wall in the apex relative to the base. Additionally, note the abnormally thickened atrial walls and inter-atrial septum. (**D**) Native T1 mapping shows normal T1 times (980 ms) while the calculation of the extracellular volume fraction (ECV) showed no interstitial expansion (ECV 18%). (**E**–**H**) Late Gadolinium enhancement images in 4, 2, 3 chambers and short axis, respectively, showing no area of hyperenhancement suggesting the absence of focal myocardial fibrosis.

Cardiac magnetic resonance (CMR) imaging confirmed LV hypertrophy involving the septal (anteroseptal basal segment 14 mm) and the apical regions (circumferential 12–13 mm), revealing a spade-like LV cavity (Figure 3). The hypertrophy of the myocardium extended beyond the LV, with a thickening of the right ventricular (RV) wall (6 mm), the atrial wall and the inter-atrial septum. Both the native T1 relaxation time (990–1000 ms) and extracellular volume (18%) were normal. A delayed contrast enhancement was not seen in any wall segment.

**Figure 4:**
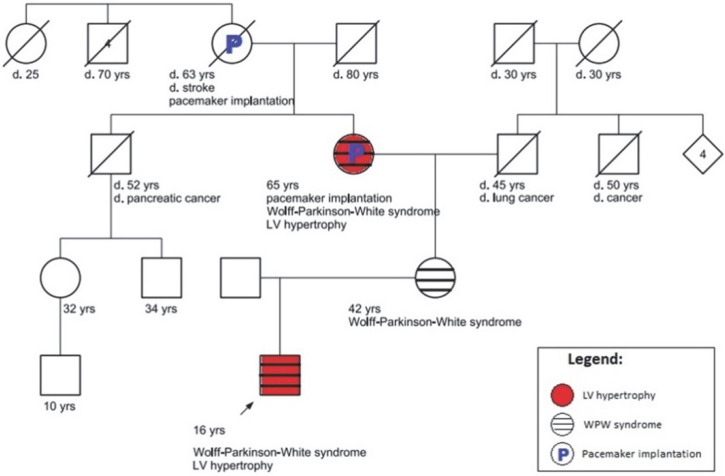
Pedigree including 4 generations. Solid black arrow indicates the proband; the solid red-filled symbol means a diagnosis of LV hypertrophy; the stripped symbol indicates subjects affected by Wolff–Parkinson–White syndrome; the “P” symbol shows the presence of an implantable pacemaker.

The patient had a strong cardiovascular family history, revealing that the patient’s maternal relatives, including his mother, grandmother and great-grandmother were known with various cardiac comorbidities (Figure 4). The patient’s mother, a 42-year-old woman, was known to have Wolff–Parkinson–White (WPW) syndrome since she was 24 years old, remaining asymptomatic. His 65-year-old maternal grandmother underwent a pacemaker (PM) implantation at 47 years old for syncope related to a third degree atrioventricular block; she had only recently been diagnosed as having non-obstructive hypertrophic cardiomyopathy (NO-HCM). The patient’s maternal great-grandmother was implanted with a PM at 45 years old. She had a history of atrial fibrillation (AF) and stroke at 55 years old and died at 63 years old with heart failure syndrome; unfortunately, no echo data were available. After evaluating the index patient, a complete cardiological workup was performed on both the mother and the grandmother.

**Figure 5:**
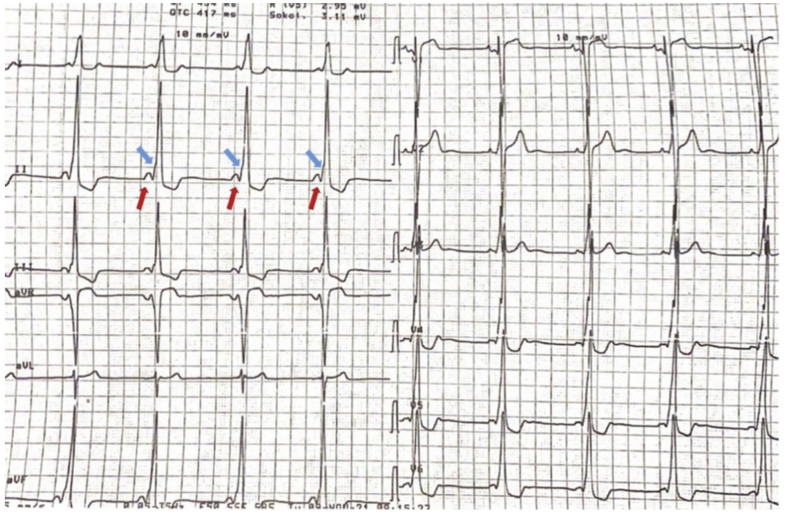
Mother’s ECG with features of WPW in sinus rhythm (short PR interval 90 ms—red arrows, and delta waves—blue arrows) and LVH voltage criteria (Sokolov–Lyon criteria of 47 mm).

The mother’s electrocardiogram showed a sinus rhythm with a very short PR interval (90 ms), broad QRS complexes (120 ms) due to a delta wave (consistent with the right medio-septal accessory pathway) as well as increased QRS voltages (Sokolov–Lyon criterion of 47 mm) with an LV strain pattern (Figure 5); however, neither the echocardiogram, nor CMR found any LV hypertrophy, with a wall thickness below 10 mm circumferentially. A significant burden (10%) of polymorphic ventricular premature complexes (PVC) as well as frequent atrial premature contractions, without any sustained arrhythmias were found with Holter monitoring. No significant bradyarrhythmia was detected.

**Figure 6:**
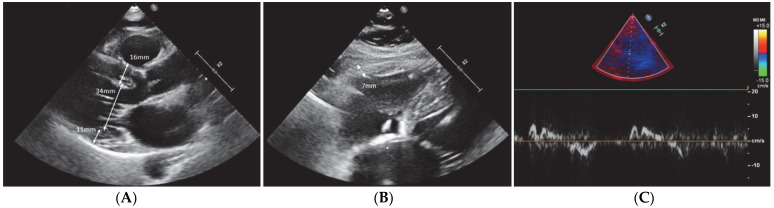
Transthoracic 2D echocardiography of the grandmother: (**A**) Parasternal long axis view showing asymmetrical LV hypertrophy (interventricular septum 16 mm; posterior wall 11 mm); (**B**) subcostal view focused on the right ventricle indicating mild RV hypertrophy (RV free wall thickness of 7 mm); (**C**) pulsed-wave tissue doppler (septal mitral annulus site) showing reduced myocardial velocities: s’ = 5.5 cm/s, and e’ = 6 cm/s.

The grandmother’s ECG showed a right ventricular apex paced QRS at the rate of 60/min on the background of an atypical atrial flutter. Older tracings demonstrated a paced QRS with retrograde P’ waves and intermittently-conducted sinus beats with QRS-T wave changes consistent with LV hypertrophy. The echocardiographic findings included biventricular hypertrophy, with asymmetrical LVH (interventricular septum 16 mm, posterior wall 11 mm, and right ventricle free wall thickness 7 mm) and no intraventricular obstruction, with a preserved LVEF but with LV longitudinal systolic dysfunction (low tissue velocities septal S’ 5.5 cm/s, and septal e’ 6 cm/s) (Figure 6). CMR could not be performed because of a non-MRI compatible cardiac device.

The presence of HCM, WPW and atrioventricular (AV) block in several family members of both sexes on the maternal line raised suspicion for an autosomal dominant genetic disorder. A next-generation sequencing genetic analysis with a cardiomyopathy dedicated gene panel (INVITAE laboratory, United States) was performed in all three family members, identifying a missense pathogenic variant (c.905G>A, Arg302Gln, heterozygous) in the *PRKAG2* gene. The p.Arg302Gln (also known as p.R302Q) is a pathogenic mutation located in coding exon 7 of the *PRKAG2* gene, and results from a G to A substitution at the nucleotide position 905, with the arginine at codon 302 being replaced by glutamine, an amino acid with highly similar properties. The mutation is a missense variant in the *PRKAG2* gene that is in a position that is highly conserved.

All three family members were diagnosed at a young age with moderate to severe arterial hypertension. The index patient and his grandmother required antihypertensive therapy at a young age. No further treatment was required for the mother. No features of skeletal myopathy or an elevated CK were found in any of the three patients.

The affected boy and his grandmother continued to remain asymptomatic under the current therapy. The mother experienced occasional transient palpitations, with an initial management by monitoring with serial ECGs, Holter and echocardiograms.

## 3. Discussions

The present case report describes an asymptomatic adolescent, with a family history of cardiac disease, in whom the incidental discovery of ECG-changes led to the diagnosis of a rare form of cardiac glycogenosis. Furthermore, a four-generation family with four affected individuals was diagnosed as having a *PRKAG2* mutation leading to various presentations which could include LVH, Wolff–Parkinson–White syndrome and/or conduction system disease.

The prevalence of the PRKAG2 syndrome is currently unknown; however, it is still rising as genetic testing is used more and more frequently in the diagnostic workup of HCM. Arad et al. reports that 29% of patients with LVH and ventricular pre-excitation were genetically confirmed as having PRKAG2 syndrome [7]; however, the prevalence is probably even higher because not all patients are associated with cardiac hypertrophy, as was described by Gollob et al. [8].

Several mutations have been identified to cause PRKAG2 syndrome, and all these have been missense mutations in the *PRKAG2* gene [9,10]. The variant (c.905G>A, p.Arg302Gln) identified in this family is the most common mutation reported in the literature. In a systematic review, including 193 PRKAG2 patients from 23 published studies, the most commonly reported mutations were C.905G>A (Arg302Gln) and c.1463A>T (Asn488Ile), with 110 and 40 cases (respectively, 57% and 21%) [11].

For many years, studies have considered the central role of the deposition of amylopectin as a major mechanism of disease in *PRKAG2* mutations-linked disease. However, Murphy et al. reported ECG temporal changes in the absence of ultrastructural or echocardiographic changes (a 100% penetrance of ECG changes before 18 years of age, as compared to a 78% penetrance of LVH after 18 years of age), as well as mitochondrial proliferation with minimal excess glycogen on skeletal muscle histology [1]. These findings suggest that deposition may not be the only mechanism of disease, and that these cellular systems might be uniquely susceptible to derangements in ATP handling. An experimental study on mice showed that cardiac hypertrophy, independently of glycogen storage, is caused by an enhanced insulin sensitivity and protein kinase B activation [12], while mutated AMPK could unbalance the phosphorylation state of cardiac troponin and myocardial contractility [13]. A study published by Liu Y et al. was able to identify unexpected histological characteristics as the presence of interstitial fibrosis and myofibrillar disarray, evidence that PRKAG2 syndrome can mimic HCM even in the histopathological analysis, and showing that fiber disarray is not a pathognomonic feature of HCM [14]. Another important histological finding that can be found is a fibrofatty replacement [15].

The most common electrocardiographic feature is a short PR interval present in 68% of patients [6]. Another essential feature is represented by advanced heart blocks, leading to premature pacemaker implantation [11] mainly in the 3rd to 4th decade of life, as was the case with the grandmother and the great-grandmother here. Murphy et al. followed a large cohort of *PRKAG2* mutation patients and found that by the end of the study period with a mean follow-up interval of 12 years, 17 of the 45 patients (38%) had pacemakers implanted, 7 for atrioventricular block, and 10 for symptomatic sinus bradycardia or chronotropic incompetence. The mean onset age for symptomatic conduction disease was 38 years (with a median of 37 years, and range of 16 to 56 years). In the systematic review of 23 studies reported by Porto et al., conduction system dysfunction had a prevalence of 44%, and PM implantation was performed in 43% of patients [11]. The findings of the presented family are in line with these data from the literature, with the first two generations of patients receiving a pacemaker in their 5th decade of life.

Supraventricular tachyarrhythmias (SVT), mainly represented by atrial fibrillation and flutter, were reported in 38% of PS patients, and a considerable proportion of them was associated with accessory pathways [1,4]. The AF onset age usually was very young, with an average of 43 ± 16 years [16]. There is evidence that the p.Arg302Gln mutation directly caused atrial damage including the intense vacuolization of cardiomyocytes and fibrosis in the samples of mutant left atrial appendage tissues obtained from individuals carrying the p.R302Q mutation compared with age-matched individuals with AF who were genotype negative [17].

In a study in which young adults with cryptogenic stroke were enrolled, cardiogenic disease genes were found (among which PRKAG2 was present), indicating that cryptogenic stroke could be caused by cardiogenic sources, even in the absence of structural heart abnormalities. The main mechanism for cerebral embolism in cardiogenic stroke is represented by dysrhythmias [18].

Sudden cardiac death (SCD) is a severe complication which occurred in 8.7% patients (mean age of death 33.4 years) without a clear trigger from a systematic review [11], and in 8% of a large, multicentric European cohort published more recently [16]; however, a recent South Asia cohort revealed a much higher rate (27%) of SCD [19]. Several etiologies are possible, from a sudden onset high degree AV block to ventricular fibrillation which can be linked to high-rate supraventricular arrhythmias and WPW or to severe LVH.

In the family presented here, most of the *PRKAG2* mutation carriers were also associated to LVH. Recently, Pena et al. published echocardiographic features in a large cohort of *PRKAG2* mutations (mostly p.Arg302Gln patients) [20], finding a mean septal thickness of 14.1 ± 4.2 mm with an asymmetrical pattern (with a mean posterior wall thickness of 12.6 ± 3.2 mm), as well as an RV wall thickness of 7.9 ± 2.9 mm. Of note, these parameters showed no differences among the patients with or without pacemakers. They described a normal LV ejection fraction but subclinical LV dysfunction with an average LV GLS of −16.4 ± 5.3%. The LV systolic dysfunction was more often detected in patients with pacemakers. No resting LV outflow tract obstruction was detected, as in our case-study family. This is in line with observations from other studies—although PRKAG2 syndrome is classically associated with severe LVH, fewer than one half of the affected subjects in a multicentric European cohort had an LV wall thickness of more than 20 mm [16]. The hypertrophy pattern may vary; some studies report concentric hypertrophy [21], as is the most common pattern in metabolic and infiltrative disorders, and other studies demonstrate an eccentric pattern [22,23,24] in patients with *PRKAG2* defects. There are some cases described in the literature of massive LV hypertrophy (with a intraventricular septal wall of 44 mm) causing myocardial ischemia due to the demand–supply mismatch of a severely hypertrophied septum [23]. A few cases present apical hypertrophy in PRKAG2 syndrome patients [25]. Regarding the myocardial tissue characterization by CMR, the findings depend on the progression of the disease. In patients at earlier stages of the disease, when late gadolinium enhancement is not present, the T1 values may be reduced, while in the advanced disease stage, T1 mapping may result in higher values caused by extracellular fibrosis [22]. About the late gadolinium enhancement burden (indicative of fibrotic scarring), only two of eight patients from a study by Ahamed H et al. presented significant myocardial enhancement, and these two individuals were also associated with marked LV hypertrophy comparing to the other six who did not express significant hypertrophy [19]. These findings are consistent with the results of the CMR exams performed on the proband and his mother, with both of them having no myocardial fibrosis.

This case also highlights the utility of genetic testing in diagnosing a patient with PRKAG2 syndrome. Not only for the involvement of the gene, but also the exact mutation that appears to influence the disease evolution in PRKAG2 syndromes. Porto et al. reported that patients with a c.905G>A mutation (such as in our family) have a greater prevalence of pre-excitation compared with c.1463A>T mutation, as well as a higher frequency of syncope and rate of PM implantation. Conversely, LVH seems to have a higher frequency in the c.1463A>T group than in c.905G>A subjects. Additionally, the most severe mutation, namely, c.1592G>A (Arg531Gln) reported until now, is characterized by an extreme early onset and a severe clinical course leading to death from cardiogenic shock within the first three months of life [26]. There is, however, a downside to genetic testing. For example, there are some variants that do not fulfill the criteria for pathogenicity and are classified as variants of unknown significance, making the diagnostic and decision-making process much more difficult, as was showed by Lopez-Sainz et al. in 30% of the patients included in their study [16].

Phenotypic heterogeneity between different families was already reported [1], but we have reported on the phenotypic variability within the same family. The particularity of this case is that the index patient already had LV hypertrophy and the features of conduction disorder (i.e., a short PR interval, but no delta waves) at 16 years old, while his mother of 42 years old only had WPW and no LVH. At a similar age, both the maternal grandmother and great-grandmother had received a pacemaker for a complete AV block. From the available documents, it appears that the grandmother developed NO-HCM after needing a pacemaker. Therefore, out of the four family members (with the exception of the great-grandmother from whom we did not have echocardiographic data available), only the mother did not develop LV hypertrophy, even though all three were diagnosed as hypertensive. Only the index patient and his grandmother needed antihypertensive treatment; thus, we can hypothesize that the early-age of occurrence and the severity of arterial hypertension accelerated the hypertrophy.

The association of high blood pressure to PS is not fully explained. We know that up to 50% of *PRKAG2* pathogenic mutation carriers were also reported to have hypertension [25].

Skeletal myopathy is much less frequent than cardiac involvement, as Sternick et al. reported an incidence of only 5.4% of cases with myopathic symptoms [27]. The skeletal muscle involvement may be associated with particular mutations, especially p.Asn488Ile [1] and p.Ser548Pro mutations [28].

Given the association between LVH and ventricular excitation and also the early onset of the disease in the male patient (compared to the women in the family), some X-linked storage disorders (e.g., Danon’s disease and Anderson–Fabry disease) needed to be excluded. Their recognition is relevant as enzyme replacement therapy is available for some of these diseases and is related to a better outcome regarding the regression of symptoms and quality of life.

There is no specific therapeutic management for *PRKAG2* mutations-related cardiomyopathy at present. However, the high rate of conduction disorders makes essential an ECG and active Holter monitoring follow-up for the early detection of AV block development as well as for early AF detection (given the stroke risk especially in older patients with LVH). Lopez-Sainz et al. described that during the median follow-up period of just 2.8 years, a number of 15% of individuals who were unaffected at the baseline went on to develop signs of the condition [16]. Due to the small number of events and to the lack of follow-up data published, risk stratification for ventricular arrhythmias remains challenging. Implantation of an implantable cardioverter defibrillator in the primary prevention of SCD in the presence of *PRKAG2* mutations does not have specific guidelines, and the HCM phenocopies are generally excluded from the SCD calculator [29]. Given the numerous life-threatening outcomes of PRKAG2 syndrome, an accurate management of its complications is required. Last, but certainly not least, genetic testing, accompanied by a focused familial screening represent useful tools for diagnosis, and this way proper genetic counseling can be offered.

## 4. Conclusions

In conclusion, molecular screening for *PRKAG2* mutations should be considered in patients who exhibit cardiac hypertrophy coexisting with ventricular pre-excitation, especially with familial inheritance. The possibility of intrafamilial phenotypic variability needs to be kept in mind during cascade screening, underlining the importance of genetic diagnostics to support a precision medicine workup.

## 5. Learning Points

- PRKAG2 syndrome represents one of the HCM genocopies and highlights the need for a high index of suspicion for these conditions in the assessment of cardiac hypertrophy.
- The presence of unexplained HCM associated with WPW syndrome and/or conduction system degeneration should lead to the suspicion of PRKAG2 syndrome.
- Genotyping can be a powerful tool for identifying patients with storage phenocopies such as PRKAG2, which can be confirmed only with genetic testing by an identification of a *PRKAG2* mutation.

## Data Availability

Not applicable.
